# Development of a spirulina feed effective only for the two larval stages of *Schistosoma mansoni*, not the intermediate host mollusc

**DOI:** 10.1186/s41182-025-00727-3

**Published:** 2025-04-02

**Authors:** Takashi Kumagai, Masaaki Miyamoto, Yurino Koseki, Yasuyuki Imai, Tomoko Ishino

**Affiliations:** 1 Department of Health Sciences, Nippon Bunri University, 1727, Ichiki, Oita-shi, Oita, 870-0397 Japan; 2 Department of Parasitology and Tropical Medicine, Institute of Science Tokyo, 1-5-45, Yushima, Bunkyo-ku, Tokyo 113-8510 Japan; 3 KYORIN, CO, LTD, 9, Shirogane-Machi, Himeji-shi, Hyogo, 670-0902 Japan; 4 Health Care Technical Group, Chiba Plant, DIC Corporation, 12, Yawata-Kaigandori, Ichihara, Chiba 290-8585 Japan

**Keywords:** *Schistosoma mansoni*, Spirulina, *Biomphalaria glabrata*, Linoleic acid

## Abstract

**Background:**

Schistosomiasis control relies primarily on mass drug administration with praziquantel. However, persistent reinfection and high treatment costs remain significant challenges. Current strategies largely overlook intermediate host molluscs and infected larvae, which are critical sources of transmission. Niclosamide, the only widely used molluscicide, is limited by its high environmental toxicity and cost, creating a need for safer and more sustainable alternatives.

**Methods:**

In this study, we investigated the effects of a spirulina-based feed derived from the cyanobacterium *Arthrospira platensis* on infected snails. Laboratory experiments were conducted to assess the impact of spirulina on cercariae release from infected snails. We further examined the safety profile of spirulina by testing its effects on both snails and Japanese medaka. Additionally, the direct effects of spirulina constituents on cercariae viability were evaluated.

**Results:**

Snails fed spirulina presented a significant reduction in cercariae output, with reductions of up to 88%. The reduction was concentration dependent and more pronounced during the early stages of infection. Spirulina had no toxic effects on either snails or Japanese medakas. Further analysis revealed that the active ingredient causing the increase in mortality in cercaria was linoleic acid, a common ingredient in both the spirulina feed and the base feed, and a direct anti-parasitic effect of linoleic acid was confirmed.

**Conclusion:**

Spirulina represents a promising, environmentally safe feed that can reduce the transmission of schistosomiasis by directly impacting schistosome larvae within infected snails and reducing the release of cercariae. This novel approach offers a sustainable and nontoxic alternative to current molluscicidal strategies and may contribute to more effective and environmentally friendly schistosomiasis control.

**Supplementary Information:**

The online version contains supplementary material available at 10.1186/s41182-025-00727-3.

## Introduction

Schistosomiasis is a neglected tropical disease that affects 250 million people worldwide; the WHO roadmap aims for “elimination of the disease as a public health problem” by 2030 [1–3]. Among the five species of schistosomes known to infect humans, *Schistosoma mansoni* is commonly found in tropical Africa and South America, while *S. haematobium* is predominantly distributed in Africa [4]. The intermediate hosts of these schistosomes are aquatic molluscs, which are widely distributed in lakes and swamps. The intermediate hosts of these schistosomes are aquatic molluscs, which serve as breeding grounds for the parasites in lakes and swamps. The infective larvae, namely, miracidia, invade these snails and are quickly converted into sporocysts. The mother sporocysts and daughter sporocysts are then followed by the formation of numerous cercariae in the larval bodies after four weeks of infection. These cercariae invade humans via percutaneous infection and become pathogenic. The primary control measure for schistosomiasis is mass drug administration (MDA) with praziquantel. However, the COVID-19 pandemic has raised concerns about reduced treatment coverage [5]. Although therapeutic agents are effective in eliminating adult worms, humans can easily become reinfected. Therefore, implementing infection prevention measures alongside treatment is considered effective [6].

One of the strategies to prevent infection is to control intermediate snails [7, 8]. In particular, the use of molluscicides is a long-established method, and the WHO has approved niclosamide as the only molluscicide [7–9]. However, niclosamide has been used for more than 20 years, and there are concerns that it is not cost effective [10] and that drug-resistant molluscs may emerge [11]. A major problem with niclosamide is its effect on nontarget organisms [12–15]. Recent environmental issues and their associated side effects, which pose threats to ecosystems and biodiversity, represent critical challenges that require resolution.

Many new molluscicides are being developed as alternatives to niclosamide. These include compounds that improve upon niclosamide, naturally derived agents with lower environmental toxicity, and plant-based extract [16, 17]. While some of these new agents have shown reduced toxicity while maintaining effectiveness, they have not yet been implemented in real-world field applications. Furthermore, ecotoxicity assessments are often conducted using zebrafish, which complicates the evaluation process [18, 19]. Achieving selective toxicity that targets only molluscs remains a significant challenge.

When we tested several commercial fish feeds as food for the intermediate host, the snail, we happened to find one that reduced the rate of cercariae infection. After preliminary tests, we selected a spirulina feed made from the dried biomass of *Arthrospira* (*Spirulina*) *platensis*.

*A. platensis* is a photosynthetic blue‒green spiral or bulbous cyanobacterium. It is rich in minerals, vitamins, pigments (carotene, phycocyanin, chlorophyll), protein (55%-70%), carbohydrates (15%-25%), and fatty acids (5%-8%) and has been used as a dietary supplement for decades [20, 21]. Spirulina is also used as a nutritional supplement for livestock and humans and has been shown to have growth-promoting, antiviral and antioxidant effects [22–24]. In previous reports, C-Phycocyanin extracted from *A. platensis* SOS13 was shown to have molluscicidal effects on *B. glabrata*, the intermediate host snail of *S. mansoni* [25]. There have also been reports that *A. platensis* extract has anthelmintic activity against sheep tape worms in vitro [26]. These results suggest that *A. platensis* influences the intermediate host snail and may also act directly on the *Schistosoma* spp., which belong to the same Platyhelminthes.

In the present study, spirulina feed was found to reduce only the number of infected *S. mansoni* larvae without affecting snail mortality. Furthermore, a direct killing effect on cercariae was observed when spirulina powder was added directly to the larvae. These results suggest a new approach for the control of schistosomiasis in which spirulina acts only on the larvae inside the snail, with greatly reduced environmental toxicity.

## Methods

### Ethics statements

All the animal experiments were performed at the Department of Parasitology and Tropical Medicine, Institute of Science Tokyo. All animal experiments were approved by the Institutional Animal Care and Use Committee of Institute of Science Tokyo (A2021-131A).

### Animals and parasites

To maintain the life cycle of *S. mansoni*, ICR mice (6 weeks old) from SLC (Hamamatsu, Japan) and the intermediate host *Biomphalaria glabrata* (Puerto Rico strain) were infected as previously described. The mice were infected by placing their tails in a tube containing 180 cercariae in tap water. The infected animals were kept in a controlled temperature and humidity environment with a 12:12 h light:dark cycle. The mice had free access to water and food in the facilities approved by the Institutional Animal Care and Use Committee of Institute of Science Tokyo (2010002C2). Humane endpoints were applied when evidence of severe pain, excessive distress, suffering, or impending death was observable in any of the animals, which were then euthanized. All mice were euthanized by CO_2_ gas at the end of the experiment. The status of all the mice was checked daily via a composite score that included vitality, secretions, fur quality, mobility, dyspnoea, ascites, neurological signs, and the ability to ingest water or food. All methods were carried out following relevant guidelines and regulations. All animal handling methods complied with the ARRIVE guidelines.

### Feed materials

**Fig. 1:**
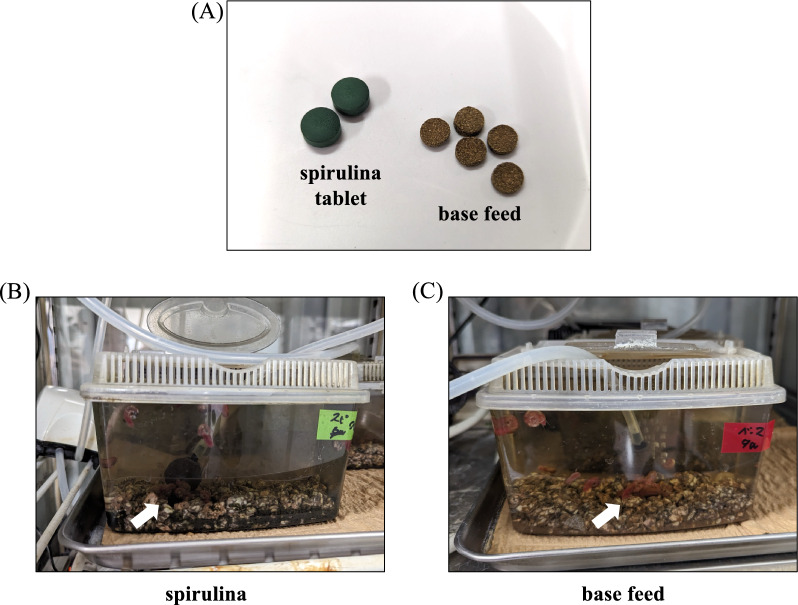
Feed used in this study. **A** Left, spirulina tablets; right, basal feed. The environment in which infected snails were used is shown. spirulina tablets (**B**), base feed (**C**). The arrows show where the snails congregated and fed

Spirulina tablets were produced by mixing 98.8% dried spirulina powder (DIC corporation, Japan), 1% calcium stearate and 0.2% fine silicon oxide into granules in a tablet press (Fig. 1A). Spirulina powder was also provided and mixed into the base feed. The base feed contains the minimum nutrients for fish feeding and is composed of 40% fish meat, 13% wheat germ, 12% soya, 7% silkworm pupae, 6% dried bakery product, 2% krill, 12% wheat flour, 3% fish oil and 4% beer yeast (Fig. 1A). The powdered material was kneaded with a small amount of water and finally heated to harden. Sizes were prepared in the range of 50–100 mg. Phycocyanin from spirulina (DIC Corporation, Japan) and linoleic acid (Sigma‒Aldrich Co. LLC, Missouri) were commercially available. Each reagent was prepared as a tablet form of feed by mixing with a base powder feed.

### Compositional analysis of feeds

Fatty acid quantification was performed via gas chromatography (GC). The sample was extracted, brought to a constant volume, and fractionated. Heptadecanoic acid was added as an internal standard, and the solvent was evaporated. Subsequently, 0.5 mol/L sodium hydroxide in a methanol solution was added for saponification. Methyl esterification was then conducted by adding a boron trifluoride methanol complex. After the reaction, hexane and saturated saline were added to form a hexane layer, which was then collected. The collected hexane layer was analysed by gas chromatography (GC) to determine the fatty acid methyl esters (FAMEs). The phycocyanin content was quantified via the AOAC method according to a standard protocol [27].

### In vivo experiments with spirulina feed on infected snails

Livers from infected mice were digested with collagenase and Actinase E (Kaken Pharmaceutical Co., Ltd., Tokyo) at 37°C for several hours, and only eggs were collected via a 35-µm diameter mesh. The eggs were hatched in tap water, and light-attracted miracidia were collected. A single snail was placed in each well of a 24-well plate, and eight miracidia were added to each well for infection. After overnight incubation, 24 snails were placed in each tank (1.4 L). The rearing was carried out in a darkroom cabinet (28°C) holding up to six aquaria filled with calcium carbonate stones and equipped with an air pump (Fig. 1B, C). The feeding conditions were as follows: 200 mg of food was added to the tank twice a week. The water in the tanks to which spirulina was added had a greenish colour but did not affect the growth of the snails. To assess the survival rate of the snails, each snail was individually placed in a 24-well plate, and the number of surviving individuals was recorded. Subsequently, the plate was exposed to light for one hour to induce cercarial shedding. After the snails were removed, a drop of potassium iodide solution was added to the remaining solution to fix the cercariae. All cercariae were counted under an inverted microscope. Data from experiments where the infection rate in the base feed group was less than 80% due to factors such as deterioration of the environmental conditions in the tank were excluded.

### Spirulina administration to cercariae in vitro

Infected snails that were passaged were exposed to light to allow the shedding of cercariae. Each feed sample was ground to powder by scraping in an agate bowl; the solution was adjusted to 0.2 mg/100 µL and diluted twofold in steps to make the administered feed. The 96-well plates were prefilled with 100 µL of the food mixture, followed by 100 µL of the cercariae mixture (15–30 cercariae), and observed under light-shielded conditions. Eight hours later, the number of cercariae was counted under an inverted microscope, and the survival rate was calculated. First, dead larvae (those that were not moving, not swollen, or whose membranes were destroyed) were counted. Larvae with only the head were considered alive if they moved. All the wells were treated with iodine‒potassium iodide solution, and the larvae were counted after all the larvae had fallen to the bottom of the wells. The survival rate was calculated by subtracting the number of dead larvae from the total number of larvae and dividing by the total number of larvae.

### In vivo experiments with spirulina fed to *Oryzias latipes* (Japanese medaka)

The ecotoxicity study with *O. latipes* was conducted following OECD guidelines (203). Specifically, *O. latipes* were kept in watertight 60 L tanks with 10 individuals per group. The watertight tanks were aerated and maintained at 26°C. Spirulina powder was added at concentrations of 100 mg/L, 50 mg/L, 25 mg/L, 12.5 mg/L, and 6.3 mg/L. A group to which no feed was added was prepared as a control. Survival was measured after 96 h without feeding anything other than the test diets. In the experiment with spirulina tablets, one tablet was equivalent to 200 mg, so the same 96-h feeding experiment was conducted with 58, 29, 15, 8, and 4 tablets in each tank.

### Statistical analysis

Student's t test was used to determine the significance of differences in means between two groups. For comparisons among three or more groups, one-way analysis of variance (ANOVA) was employed, followed by the Tukey test for post hoc analysis of significant differences. All the statistical analyses were conducted via SPSS version 29 (SPSS Inc., Chicago, IL, USA).

## Results

### Effects of spirulina on *S. mansoni*-infected snails

**Fig. 2:**
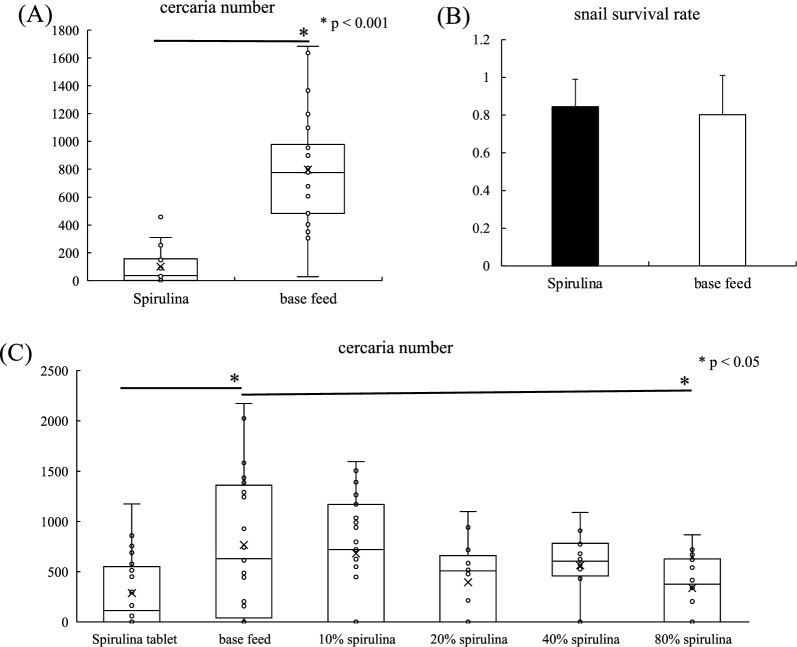
Reduction in the number of cercariae released from infected snails by spirulina feed. **A** In each column, the dots indicate the number of cercariae released from infected snails that were fed, and the boxes indicate the 25–75% range (one of three experiments is shown). Student’s t test was used to determine the significance of differences in means between two groups. **B** Each column shows the survival rate of infected snails treated with the feed (mean of three experiments). **C** In each column, the dots indicate the number of cercariae when infected snails were given feed containing different concentrations of spirulina, and the boxes indicate the 25–75% range. One-way ANOVA was employed, followed by the Tukey test for post hoc analysis of significant differences

We identified an ingredient in the feed used to feed *B. glabrata* that unexpectedly reduced the number of cercariae produced. We investigated the components of the feed and found spirulina to be effective and conducted this experiment. When *B. glabrata* infected with *S. mansoni* miracidia were treated with spirulina tablets (200 mg) twice a week for 6 weeks, a significant difference in the number of cercariae released was detected (Fig. 2A). This experiment was repeated three times, with a maximum reduction of 88%. No adverse effects on snail survival were observed, and survival rates were comparable to those of the base feed group (Fig. 2A, B). The effect of incorporating powdered spirulina into the base feed on cercariae development was evaluated in relation to spirulina concentration, revealed a concentration-dependent reduction in the cercaria release (Fig. 2C). These findings suggest that spirulina does not exert toxicity on infected snails but specifically inhibits the development of *S. mansoni*.

### Effects of spirulina on the course of infection

**Fig. 3:**
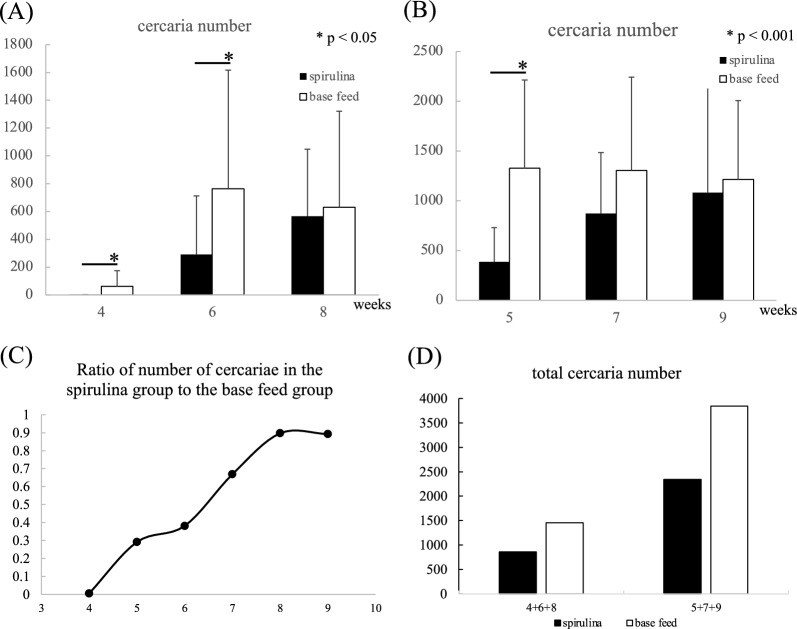
Effects of spirulina on reducing cercariae counts per week of infection. **A** Each column shows the mean number of cercaria released from groups of infected snails collected at 4, 6 and 8 weeks from feed-treated infected snails. Student's t test was used to determine the significance of differences in means between two groups. **B** Each column shows the mean number of cercaria released from groups of infected snails collected at 5, 7 and 9 weeks from feed-treated infected snails. Student's t test was used to determine the significance of differences in means between two groups. **C** Ratio of the mean number of cercariae released by spirulina administration in the base feed. **D** Columns showing the total number of cercariae recovered from each weekly infected group

The effect of spirulina on larval development was observed at each post-infection stage. The experiment was conducted in two phases due to limitations on the number of available aquaria. The number of cercariae was examined at 4, 6, and 8 weeks in the first phase, and at 5, 7, and 9 weeks in the second phase (Fig. 3A, B). The number of cercariae was significantly reduced in the spirulina-treated group during early post-infection stages, but this reduction diminished over time. The percentage of cercariae in the spirulina-treated group, relative to the base feed group, increased over time (Fig. 3C). These findings suggest that spirulina inhibits cercariae development or reduces their number during early infection stages. Despite the diminished effect over time, the total number of released cercariae was still lower in the spirulina-treated group, indicating a potential suppressive effect (Fig. 3D).

### In vivo effects of phycocyanin on infected snails

**Fig. 4:**
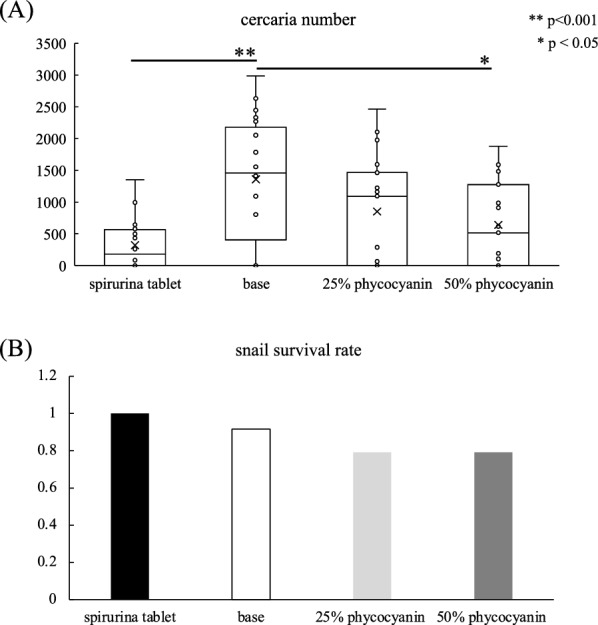
Reduction in the number of cercariae released from infected snails by phycocyanin. **A** In each column, the dots indicate the number of cercariae released from infected snails that were fed, and the boxes indicate the 25–75% range. One-way ANOVA was employed, followed by the Tukey test for post hoc analysis of significant differences. **B** Each column shows the survival rate of infected snails treated with the feed

Previous studies have reported that phycocyanin, a component of spirulina, exhibits toxicity to snails [25]. Thus, the observed effects of spirulina on schistosomes in this study were hypothesized to be associated with phycocyanin. As the spirulina tablets used in this study contained approximately 10% phycocyanin (Table 1), we conducted additional experiments using feeds supplemented with 25% and 50% purified phycocyanin. The feed containing 25% phycocyanin had no detectable effect on cercariae release (Fig. 4A). In contrast, the 50% phycocyanin feed reduced cercariae numbers, though it was less effective than spirulina tablets. A slight decrease in snail survival was also observed (Fig. 4B). These results suggest that phycocyanin is not the primary factor responsible for spirulina’s cercariae-reducing effect.

**Table 1:** Phycocyanin content in feeds

Phycocyanin	Base feed	Spirulina tablet
C-Phycocyanin (%)	0.0	6.8
Allophycocyanin (%)	0.0	2.6
Total (%)	0.0	9.4

### Effect of spirulina on cercariae in vitro

**Fig. 5:**
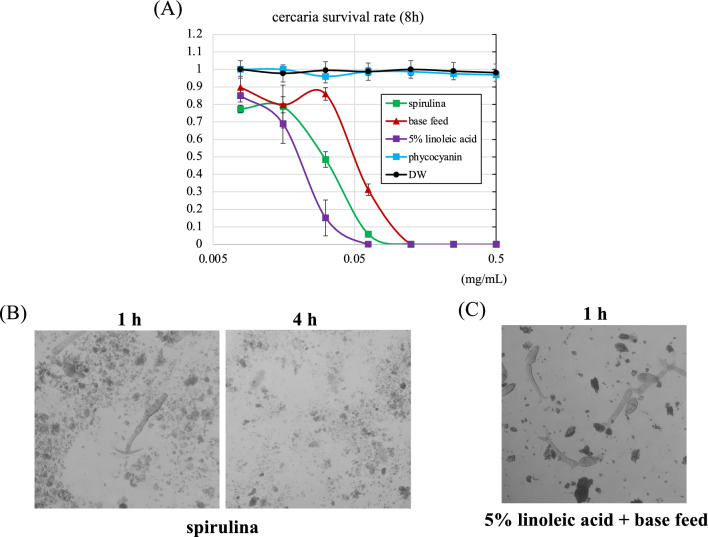
Direct effects of spirulina and linoleic acid on cercariae. **A** Concentration-dependent effects of each feed on cercariae. **B** Photograph showing morphological changes at 1 and 4 h after the administration of 0.125 mg/ml spirulina to cercariae. **C** Photograph showing morphological changes at 1 h after the administration of 5% linoleic acid + base feed to cercariae

The direct cercaricidal activity of spirulina powder was evaluated in vitro. The results revealed that spirulina powder killed cercariae in a concentration-dependent manner after 8 h of exposure (LC50 ≒ 28 µg/ml) (Fig. 5A). When a lethal dose of 0.125 mg/ml was administered, living cercariae were attracted to spirulina within one hour, and they subsequently died four hours after exposure (LC50 ≒ 49 µg/ml) (Fig. 5B). Unfortunately, the base feed used in this study also had a killing effect on cercariae, although it was weaker than that from spirulina (Fig. 5A). Compositional analysis showed that both samples contained high levels of fatty acids (Table 2). Given that linoleic acid has been reported to attract cercariae and induce tail loss, a feed supplemented with 5% linoleic acid was prepared, assuming that this effect was attributed to linoleic acid (LC50 ≥ 20 µg/ml). This feed, at a concentration of 0.125 mg/ml, killed cercariae more rapidly than spirulina did (Fig. 5C). Phycocyanin showed no toxicity to cercariae at the concentrations tested in this study (Fig. 5A).

**Table 2:** Fatty acid content in feeds

Fatty acid		Base feed (100 g)		Spirulina tablet (100 g)	
		Content in fatty acids (%)	Weight (g)	Content in fatty acids (%)	Weight (g)
14:0	Myristic acid	3.1	0.20	–	–
15:0	Pentadecanoic acid	0.3	0.02	–	–
16:0	Palmitic acid	20.5	1.30	38.4	2.44
16:1	Palmitoleic acid	3.5	0.22	5.0	0.32
16:2	Hexadecadienoic acid	0.4	0.03	0.7	0.04
17:0	Heptadecanoic acid	0.3	0.02	0.2	0.01
17:1	Heptadecenoic acid	0.2	0.01	0.2	0.01
18:0	Stearic acid	5.0	0.32	1.6	0.10
18:1	Oleic acid	21.5	1.37	2.2	0.14
18:2n-6	Linoleic acid	17.2	1.09	19.7	1.25
18:3n-6	γ-Linolenic acid	0.2	0.01	23.1	1.47
18:3n-3	a-Linolenic acid	4.0	0.25	0.3	0.02
18:4n-3	Octadecatetraenoic acid	1.0	0.06	–	–
20:0	Arachidic acid	0.4	0.03	–	–
20:1	Eicosenoic acid	2.4	0.15	–	–
20:2n-6	Eicosadienoic acid	0.1	0.01	0.3	0.02
20:3n-6	Dihomo-γ-linolenic acid	–	–	0.3	0.02
20:4n-6	Arachidonic acid	0.7	0.04	–	–
20:4n-3	Eicosatetraenoic acid	0.3	0.02	–	–
20:5n-3	Eicosapentaenoic acid	5.2	0.33	0.1	0.01
21:5n-3	Heneicosapentaenoic acid	0.2	0.01	–	–
22:0	Behenic acid	0.2	0.01	–	–
22:1	Docosenoic acid	2.5	0.16	–	–
22:5n-6	Docosapentaenoic acid (n-6)	0.2	0.01	–	–
22:5n-3	Docosapentaenoic acid (n-3)	1.1	0.07	–	–
22:6n-3	Docosahexaenoic acid	5.5	0.35	–	–
24:0	Lignoceric acid	0.2	0.01	–	–
24:1	Tetracosenoic acid	0.5	0.03	–	–
	Unidentified	3.3	0.21	8.1	0.51

### In vivo effects of linoleic acid on cercariae development in infected snails

To verify the in vivo effect of linoleic acid, the experiment was conducted again using infected snails. No inhibitory effect on larval development was observed with the new feed containing 5% linoleic acid (Fig. S1).

### Assessment of environmental toxicity in Japanese medaka

To evaluate the environmental impact of spirulina on non-molluscan organisms, a toxicity assessment was conducted on Japanese medaka (*Oryzias latipes*) based on OECD guideline 203. Both spirulina powder and tablet formulations showed no toxicity to Japanese medaka (Table 3). The water pH remained stable (7.0–7.6 for powder, 6.9–7.9 for tablets), with no significant environmental changes.

**Table 3:** Impact of spirulina tablets and powder on Japanese medaka

Spirulina powder	Japanese medaka number		pH				
Concentration	0 h	96 h	0 h	24 h	48 h	72 h	96 h
100 mg/L	10	10	7.4	7	7.1	7.3	7.5
50 mg /L	10	10	7.6	7.1	7.3	7.5	7.5
25 mg /L	10	10	7.6	7.1	7.3	7.4	7.5
12.5 mg /L	10	10	7.6	7.3	7.4	7.5	7.6
6.3 mg /L	10	10	7.7	7.4	7.5	7.5	7.6
0	10	10	7.7	7.6	7.5	7.5	7.5

Spirulina tablets	Japanese medaka number		pH				
Quantity (mg/ml)	0 h	96 h	0 h	24 h	48 h	72 h	96 h
58 (205.1)	10	10	7.9	6.9	7.1	7.3	7.4
29 (102.5)	10	10	7.9	7	7.2	7.4	7.4
15 (53)	10	10	7.9	7.2	7.3	7.5	7.5
8 (28.3)	10	10	7.9	7.4	7.5	7.6	7.6
4 (14.1)	10	10	7.9	7.6	7.6	7.7	7.7
0 (0)	10	10	7.9	7.8	7.8	7.8	7.8

## Discussion

The results of this study indicate that spirulina could be considered a candidate for an environmentally safe schistosomicide. This hypothesis was inspired by the significant reduction in cercaria release when the feed was changed in *S. mansoni*-infected snails. Feeding spirulina to infected snails reduced cercariae shedding by 88%. This dose-dependent effect indicated that spirulina either reduced the number of cercariae or inhibited their development. The next experiment examined cercariae shedding in groups divided by infection week after spirulina feeding. The results revealed a marked difference in the number of larvae in the group with the earliest infection, as the infection progressed, the difference from the base feed decreased. It was speculated that this result was not due to a simple delay in the development of cercaria but rather to the number of sporocysts in the early stages of infection. Spirulina treatment reduced the total number of larvae released, suggesting that reducing the number of larvae released is at least effective in preventing subsequent infection of mammals, including humans.

Interestingly, there was no change in the survival rate of the spirulina-fed snails. Unlike conventional molluscicides, spirulina reduces schistosomes without affecting molluscs, representing a novel approach. In particular, if it does not affect molluscs, one might imagine that its ecotoxicity to other organisms would be quite low [28]. Spirulina did not cause direct toxicity in Japanese medaka (*Oryzias latipes*). As spirulina is widely used as a dietary supplement for tropical fish, livestock, or humans, it is likely to have few adverse effects on vertebrates. This would be an advantage when spraying spirulina in areas where schistosomiasis is endemic.

Spirulina comprises a very large number of components. Further studies are needed to identify the active components responsible for this effect. The most characteristic component of spirulina is phycocyanin, which has been shown to kill snails in previous studies [25]. As the spirulina tablets used in this study contained approximately 10% phycocyanin, the isolated phycocyanin was mixed with the basal diet to produce two concentrations, 25% and 50%. The 50% phycocyanin feed reduced the number of cercariae, but the effect was less than that of spirulina alone. It also slightly reduced the survival rate of the snails, and its potential toxicity to snails was observed. These results suggest that phycocyanin alone in feed is not an essential component for cercariae reduction and is not suitable for ecotoxicity-suppressed schistosome-killing feed via this strategy.

An in vitro cercariae killing test was carried out to determine the effect of the product on cercariae released into water. The results showed that both spirulina and base feed had a killing effect on cercariae. Spirulina contains a large proportion of fatty acids (30%) [29]. In fact, spirulina tablets contain three notable fatty acids: palmitic acid, linoleic acid and gamma-linolenic acid. Schistosomes are unable to synthesize fatty acids de novo and require uptake from the host [30]. In particular, Sm25 and Sm14, as fatty acid-binding proteins, have been reported to play a role in this process by binding to palmitic and linolenic acids [31, 32]. However, linoleic acid is known to attract *S. mansoni* cercariae and induce tail loss [33–37]. In the present study, the linoleic acid content of the base feed was slightly greater than that of the spirulina feed at 1.5%. Furthermore, a base feed with a relatively high concentration of linoleic acid was prepared, which had a relatively strong killing effect. These findings confirm that linoleic acid effectively kills cercariae. However, the spirulina feed with a lower linoleic acid content was observed to kill approximately twice as much as the base feed. These findings suggest that spirulina also has a linoleic acid-independent killing effect on cercaria. This means that spirulina feeds with linoleic acid can be expected to provide further benefits. Phycocyanin, on the other hand, was not toxic to cercariae.

Loss of cercariae tails by linoleic acid has been reported to be caused by an influx of calcium ions [34]. Cercariae shed their tails during host invasion and penetrate the skin. Within the skin, linoleic acid has also been reported to play an important role in signalling [38], and linoleic acid stimulation has been implicated in the uptake of molecules within the skin [39]. Thus, rather than being toxic to cercariae, linoleic acid is a necessary factor in shifting developmental processes. The present study successfully exploited this process and killed the cercariae by detaching the tail in the environment. On the other hand, a correlation between linoleic acid levels and resistance to schistosome infection in intermediate host snails has recently been reported [40]. Although this seems to contradict the present study, the fact that there are no reports of linoleic acid feeding being involved in the acquisition of resistance suggests that further investigation is needed in this area of research.

This study has several limitations. First, the effects of spirulina components, except for linoleic acid and phycocyanin, were not evaluated, leaving the specific molecules responsible for the main effects unidentified. Although linoleic acid exhibited activity against cercariae, it did not account for all in vitro effects, nor did it contribute to the in vivo effects observed. Therefore, further investigation is required to identify the active components in spirulina. Second, the toxicity assessment was limited to *Biomphalaria glabrata* and Japanese medaka, which are standard indicator organisms according to OECD guidelines. Although additional toxicity assessments using other species are necessary, spirulina is generally recognized as safe, suggesting minimal toxicity at the concentrations used in this study. Future studies will focus on identifying the active molecules, conducting detailed microscopic examinations of snail tissues, and evaluating the effects of spirulina on sporocyst larvae.

In conclusion, spirulina significantly reduced *S. mansoni* cercariae without affecting the survival of snails or Japanese medaka, suggesting its potential as an environmentally safe schistosomicide. Further investigation is required to identify which components of spirulina are responsible for the cercariae-killing effect. The combination of spirulina and linoleic acid may be effective in the fight against cercariae. Spirulina has the potential to become a new type of strategy that does not cause environmental toxicity, and future work is expected to elucidate its mechanism and apply it in the field.

## Supplementary Information

Supplementary material 1.

## Data Availability

No datasets were generated or analysed during the current study.

## Abbreviations

AOAC: Association of official analytical chemists
GC: Gas chromatography
MDA: Mass drug administration
OECD: Organisation for Economic Cooperation and Development
WHO: World Health Organization
