# Supplementary material for: Development of a spirulina feed effective only for the two larval stages of Schistosoma mansoni, not the intermediate host mollusc
Source: Trop Med Health. 2025 Apr 2;53:46. doi: 10.1186/s41182-025-00727-3 (PMC11963688; doi:10.1186/s41182-025-00727-3)
Supplement: Supplementary file 1 — Supplementary material 1. [file 41182_2025_727_MOESM1_ESM.pdf]

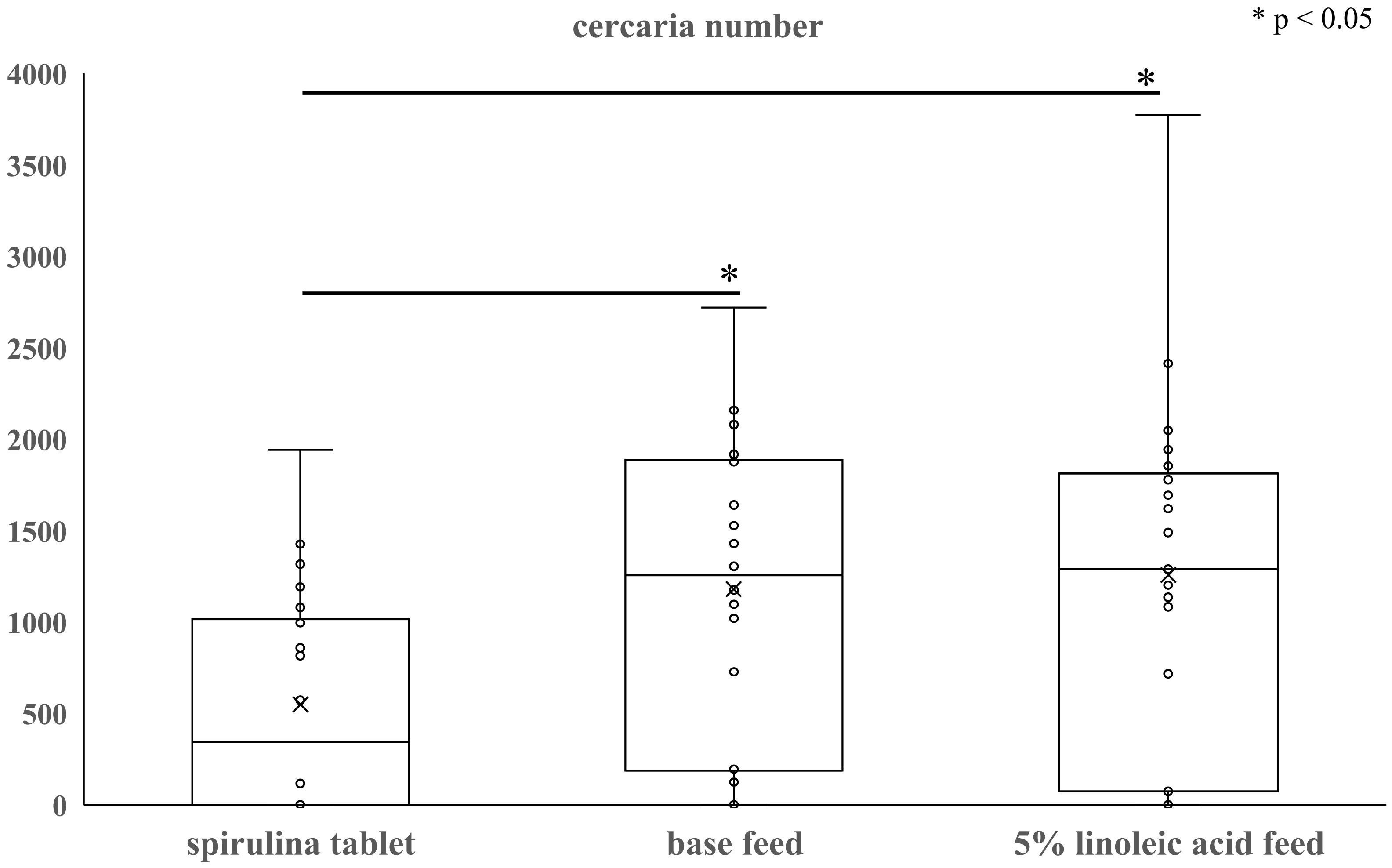

Fig. S1 No effect in the number of cercariae released from infected snails by 5% linoleic acid feed. In each column, the dots indicate the number of cercariae released from infected snails that were fed, and the boxes indicate the 25%-75% range. One-way ANOVA was employed, followed by the Tukey test for post-hoc analysis of significant differences.
